# Exosomes from Human iPSC-Derived Retinal Organoids Enhance Corneal Epithelial Wound Healing

**DOI:** 10.3390/ijms25168925

**Published:** 2024-08-16

**Authors:** Si Hyung Lee, Jung Woo Han, Jin Young Yang, Jungmook Lyu, Hyo Song Park, Ji Hong Bang, Yeji Kim, Hun Soo Chang, Tae Kwann Park

**Affiliations:** 1 Department of Ophthalmology, College of Medicine, Soonchunhyang University, Cheonan 31151, Republic of Korea; sieh12@schmc.ac.kr (S.H.L.); 106236@schmc.ac.kr (J.W.H.); 124533@schmc.ac.kr (H.S.P.); 2 Department of Ophthalmology, Soonchunhyang University Bucheon, 170, Jomaru-ro, Bucheon 14584, Republic of Korea; 3 Laboratory of Molecular Therapy for Retinal Degeneration, Soonchunhyang University Bucheon Hospital, Bucheon 14584, Republic of Korea; roswellgirl111@gmail.com (J.Y.Y.); yeiji77@naver.com (Y.K.); 4 Department of Medical Science, Myung-Gok Eye Research Institute, Konyang University, Daejeon 32992, Republic of Korea; lyujm@konyang.ac.kr; 5 Department of Interdisciplinary Program in Biomedical Science, Soonchunhyang Graduate School, Soonchunhyang University Bucheon Hospital, Bucheon 14584, Republic of Korea; jihong325@naver.com (J.H.B.); hschang@sch.ac.kr (H.S.C.); 6 Department of Microbiology, Soonchunhyang University College of Medicine, Cheonan 31151, Republic of Korea

**Keywords:** corneal epithelial wound healing, exosomes, human induced pluripotent stem cells, retinal organoids

## Abstract

This study investigated the therapeutic effects of exosomes derived from human-induced pluripotent stem cell (hiPSC)-derived retinal organoids (ROs) on corneal epithelial wound healing. Exosomes were isolated from the culture medium of the hiPSC-derived ROs (Exo-ROs) using ultracentrifugation, and then they were characterized by a nanoparticle tracking analysis and transmission electron microscopy. In a murine model of corneal epithelial wounds, these exosomes were topically applied to evaluate their healing efficacy. The results demonstrated that the exosome-treated eyes showed significantly enhanced wound closures compared with the controls at 24 h post-injury. The 5-ethyl-2′-deoxyuridine assay and quantitative reverse transcription polymerase chain reaction revealed a substantial increase in cell proliferation and a decrease in inflammatory marker contents in the exosome-treated group. The RNA sequencing and exosomal microRNA analysis revealed that the Exo-RO treatment targeted various pathways related to inflammation and cell proliferation, including the PI3K-Akt, TNF, MAPK, and IL-17 signaling pathways. Moreover, the upregulation of genes related to retinoic acid and eicosanoid metabolism may have enhanced corneal epithelial healing in the eyes treated with the Exo-ROs. These findings suggest that hiPSC-derived RO exosomes could be novel therapeutic agents for promoting corneal epithelial wound healing.

## 1. Introduction

The cornea is a transparent vascular structure of the eye that is exposed to the external environment and its integrity and transparency must be maintained to preserve visual function [1]. The corneal epithelium, located at the outermost cornea, serves as a physical barrier, and disruption of its integrity may lead to subsequent infection and stromal ulceration, which can progress to scarring or perforation [2,3]. Current treatments for corneal epithelial wounds mainly consist of supportive care, including lubrication and bandage contact lenses; however, these treatments often are ineffective for persistent and recalcitrant epithelial wounds [4]. Thus, there is a great need to develop novel treatments for more severe and persistent corneal epithelial defects.

Exosomes are 30–150-nm-diameter extracellular vesicles released by most types of cells [5,6,7]. These vesicles are secreted from cells under both normal and pathological conditions. Exosomes consist of bioactive molecules for intercellular communication, including proteins, lipids, mRNA, and microRNA. Upon reaching their target cells, exosomes fuse to cell membranes to deliver these bioactive molecules, thereby inducing phenotypic changes in the recipient cells [8]. This process enables exosomes to play crucial roles in combating inflammation, promoting angiogenesis, and aiding tissue repair [9,10,11].

Recently, exosomes derived from mesenchymal stem cells (MSCs) have demonstrated the ability to promote corneal epithelial wound healing in vitro and in vivo [4,12]. However, because MSCs are mesodermal, whereas corneal tissue is of ectodermal origin, exosomes derived from MSCs may have limited value as agents for treating corneal disorders. Enhanced therapeutic effects may be anticipated when exosomes derived from ectodermal cells are used as therapeutic agents.

In this context, we isolated exosomes from the culture medium of human-induced pluripotent stem cell (hiPSC)-derived retinal organoids (ROs), and then we investigated their therapeutic potential in corneal epithelial wound healing in vivo. We also investigated the underlying mechanisms by which exosomes derived from ROs (Exo-ROs) exert therapeutic effects on corneal epithelial wound healing by analyzing both the exosomal and total miRNA from corneal tissues.

## 2. Results

### 2.1. Uptake of Exosomes Derived from ROs by Mouse Corneas

Exosomes from a conditioned medium of hiPSC-derived ROs at day 60 were successfully isolated, and their size distributions were analyzed using a nanoparticle tracking analysis (NTA), which showed an average exosome diameter of 149.5 ± 2.9 (range, 30–550) nm with peak diameter of 72 ± 1.8 nm (Supplementary Figure S2A). The concentration of exosomes used in this study was 1.07 × 10^11^ ± 7.87 × 10^8^ particles/mL. The transmission electron microscopy (TEM) revealed round, cup-shaped exosomes with an average diameter of approximately 110 nm (Supplementary Figure S2B). Western blotting of the exosome markers [13] CD9, CD63, and CD81 showed stronger band intensities for the isolated exosomes compared with the crude precipitates of the RO-conditioned medium (Supplementary Figure S2C), and none of these were detected in the phosphate-buffered saline (PBS). β-Actin and calnexin, which are intracellular proteins that are negative markers for exosomes [13], were absent from the isolated exosomes.

**Figure 1:**
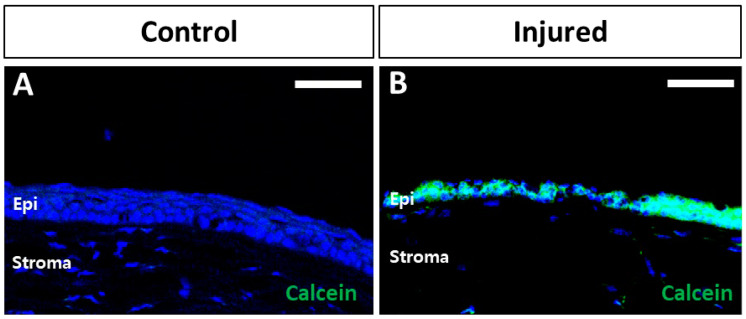
Retinal organoid (RO)-derived exosome (Exo-RO) uptake by the control and wounded corneas. The calcein (green)-labeled Exo-ROs were instilled immediately after the injury to examine the uptake of the Exo-ROs by the mouse corneal epithelial cells in vivo. (**A**) Minimal or no calcein was detected in the uninjured control corneas. (**B**) A strong calcein signal was observed in the wounded corneal epithelium (scale bar = 75 µm).

At 3 h after the calcein-labeled exosome instillation, intense green fluorescence was detected in the injured cornea epithelium compared with the uninjured control cornea (Figure 1). This finding indicated that corneal epithelial injury promoted the uptake of the Exo-ROs, whereas the exosome uptake was limited in the corneas with intact epithelial cells. Thus, the Exo-RO uptake was enhanced in the injured corneal epithelium.

### 2.2. RO-Derived Exosomes Enhanced Epithelial Wound Healing

**Figure 2:**
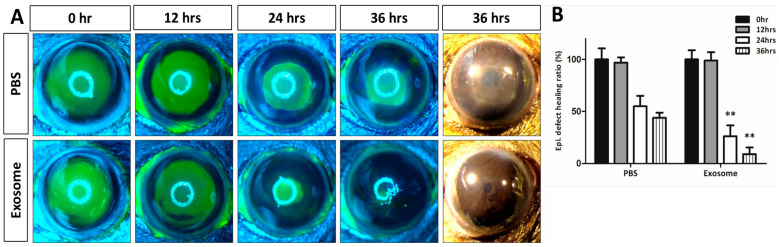
Effects of the retinal organoid (RO)-derived exosomes on corneal epithelial wound healing. (**A**) Fluorescein-stained and gross images of a 2 mm epithelial defect in a mouse cornea from the initial injury until 12, 24, and 36 h after treatment with either RO-derived exosomes or a vehicle (phosphate-buffered saline (PBS)). Mild stromal haziness was evident in the vehicle-treated cornea, and the exosome-treated cornea exhibited relatively clear stroma at 36 h after treatment. (**B**) The exosome-treated cornea exhibited superior epithelial wound healing compared with the vehicle-treated control. *n* = 5 for each group. ** *p* < 0.001, by Student *t*-test.

To evaluate the therapeutic effects of the Exo-ROs on corneal epithelial wound healing, either Exo-ROs or vehicles (PBS) were instilled immediately after injury in mouse corneas in the treatment and control groups, respectively. Although there was no significant difference in the epithelial healing ratio at 12 h after treatment, the wound-healing ratio was significantly greater in the Exo-RO-treated group at 24 and 36 h after treatment compared with the vehicle-treated group (*p* < 0.001; Figure 2). Moreover, the gross photographs of the Exo-RO-treated corneas showed relatively clear stroma at 36 h after treatment, whereas the vehicle-treated corneas showed generalized stromal haziness (Figure 2A). The hematoxylin-eosin stained images showed similar results (Supplemenatary Figure S3).

### 2.3. RO-Derived Exosomes Promote Wound Healing by Cell Proliferation and Immunosuppression

**Figure 3:**
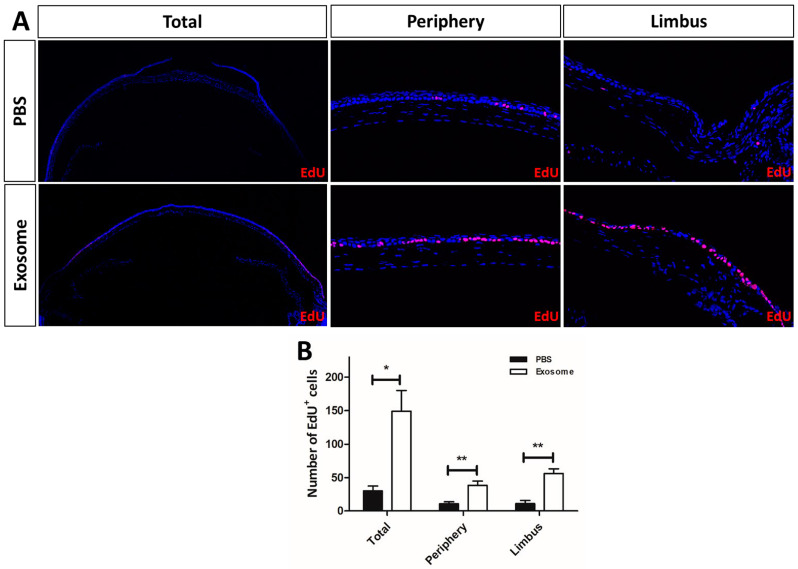
Cross-sectional images of the 5-ethyl-2′-deoxyuridine (EdU) labeling of the vehicle-treated and retinal organoid (RO)-derived exosome-treated corneas. (**A**) EdU labeling: overall and at the periphery and limbus of the vehicle and exosome-treated corneas. (**B**) The exosome-treated cornea contained significantly more EdU^+^ cells compared with the vehicle-treated cornea. *n* = 5 for each group. PBS, phosphate-buffered saline. * *p* < 0.05 and ** *p* < 0.001, by Student *t*-test.

The mechanism underlying the enhanced wound-healing process was investigated by a 5-ethyl-2′-deoxyuridine (EdU) analysis to compare the cell proliferative activity between the Exo-RO- and vehicle-treated corneas. At 24 h after treatment, the numbers of EdU-positive cells in the periphery and limbal areas of the corneal epithelium were significantly increased in the Exo-RO-treated corneas compared with the vehicle-treated corneas (Figure 3). Moreover, these proliferative signals were stronger in the basal epithelial layer, implying that treatment with the Exo-ROs accelerated the wound healing by increasing cell proliferation.

**Figure 4:**
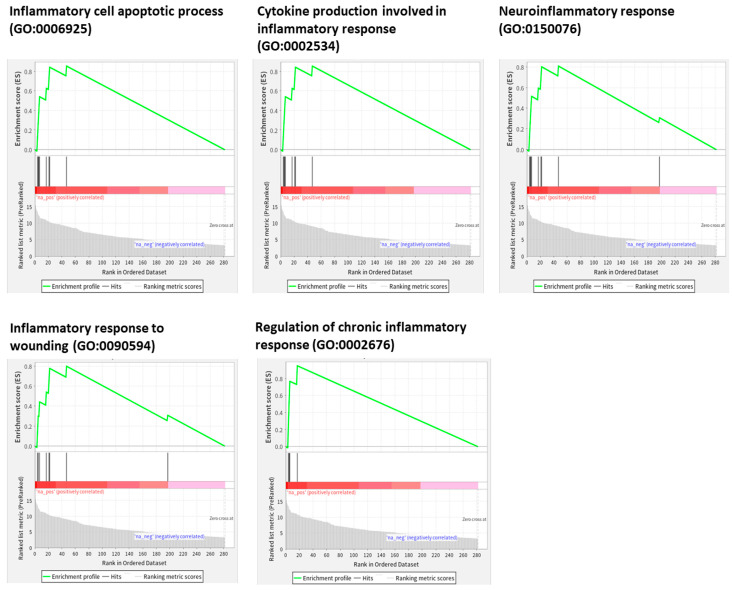
Enrichment plots of the exosomal 272 abundant miRNAs (normalized cpm ≥ 10) significantly enriched (false discovery rate [FDR] of *q* < 0.05) for the target gene ontologies. The exosomal miRNA sequencing revealed that the miRNAs mainly related to modulating inflammatory processes were significantly enriched (FDR *q* < 0.001) and mainly ranked in the top 20 in terms of abundance.

**Figure 5:**
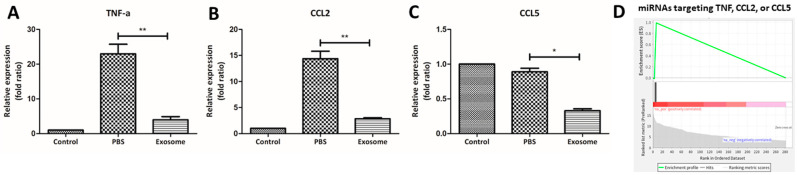
Quantitative analysis of the inflammatory cytokines from the vehicle-treated and retinal organoid (RO)-derived exosome-treated corneas at 36 h after injury. A quantitative reverse transcription (qRT)-polymerase chain reaction (PCR) analysis of the inflammatory cytokines showed that the RO-derived exosome-treated corneas contained significantly lower levels of (**A**) TNF-α, (**B**) CCL2, and (**C**) CCL5 compared with the vehicle-treated corneas. (**D**) The significantly enriched miRNAs related to TNF-α, CCL2, and CCL5 are shown in an enrichment plot. PBS, phosphate-buffered saline. *n* = 5 for each group. * *p* < 0.05 and ** *p* < 0.001, by Student *t*-test.

Next, we explored whether the Exo-RO treatment had immunomodulatory effects during wound healing by analyzing the miRNA contents of the Exo-ROs. A gene-set enrichment analysis (GSEA) of 272 miRNAs detected at least 10 cpm for 17 gene ontology (GO) terms, including “inflammatory” and “epithelial cell” (Supplementary Tables S1 and S2). As shown in Figure 4, the abundant miRNAs were significantly enriched (false discovery rate of *q* < 0.001) in “inflammatory cell apoptotic process”, “cytokine production involved in inflammatory response”, “neuroinflammatory response”, “inflammatory response to wounding”, and “regulation of chronic inflammatory response”. The miRNAs targeting the genes in these biological processes mainly ranked in the top 20 in terms of abundance. Furthermore, we compared the expression levels of the representative inflammatory cytokines, including TNF-α, CCL2, and CCL5, between the Exo-RO- and vehicle-treated corneas by quantitative reverse transcription (qRT)-polymerase chain reaction (PCR) at 36 h after treatment (Figure 5A–C). Compared with the vehicle-treated corneas, the Exo-RO-treated corneas had significantly lower expression levels of all three inflammatory cytokines (*p* < 0.001 for TNF-α and CCL2; *p* = 0.024 for CCL5), demonstrating that the anti-inflammatory effects of the Exo-ROs played a role in enhancing corneal epithelial wound healing. Moreover, the miRNAs related to these three cytokines were significantly enriched, as shown by the GO enrichment analysis (Figure 5D).

### 2.4. Differential Expression of the Genes in the Corneas Treated with Exo-ROs

**Figure 6:**
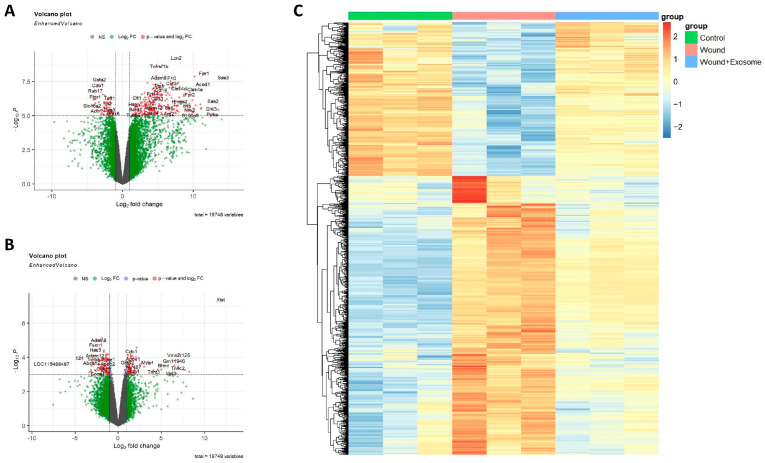
Volcano plots and a heatmap of the differentially expressed genes (DEGs) in the control, phosphate-buffered saline (PBS)-treated, and retinal organoid (RO)-derived exosome (Exo-RO)-treated groups. The volcano plots show (**A**) the DEGs between the controls and the corneal injury model with phosphate-buffered saline (PBS) treatment and (**B**) the DEGs between the PBS-treated and Exo-RO-treated groups. (**C**) Of the DEGs, 413 were upregulated and 204 were downregulated in the PBS-treated group. These DEGs were significantly reversed by treatment with the Exo-ROs.

To examine the actual biological changes occurring in the corneal tissues after Exo-RO treatment, total RNA sequencing was performed and the groups were compared. A comparison of the controls and corneal injury model with a PBS-treated group showed that 1444 genes were upregulated, whereas 954 genes were downregulated (average log_2_[CPM] > 1, |log_2_[fold change]| > 1, and FDR *q* < 0.05) in the PBS-treated group (Figure 6A). A comparison of the Exo-RO- and PBS-treated groups showed that 301 and 762 genes were up- and downregulated, respectively, in the PBS-treated group (average log_2_[CPM] > 1, |log_2_[fold change]| > 1, and *p* < 0.05; Figure 6B). The DEGs are listed in Supplementary Table S3. Of these DEGs, 413 were upregulated in the PBS-treated group. This upregulation was significantly reversed by the Exo-RO treatment (Figure 6C). Conversely, 204 genes with reduced expression in the PBS-treated group were significantly increased in the Exo-RO-treated group.

**Figure 7:**
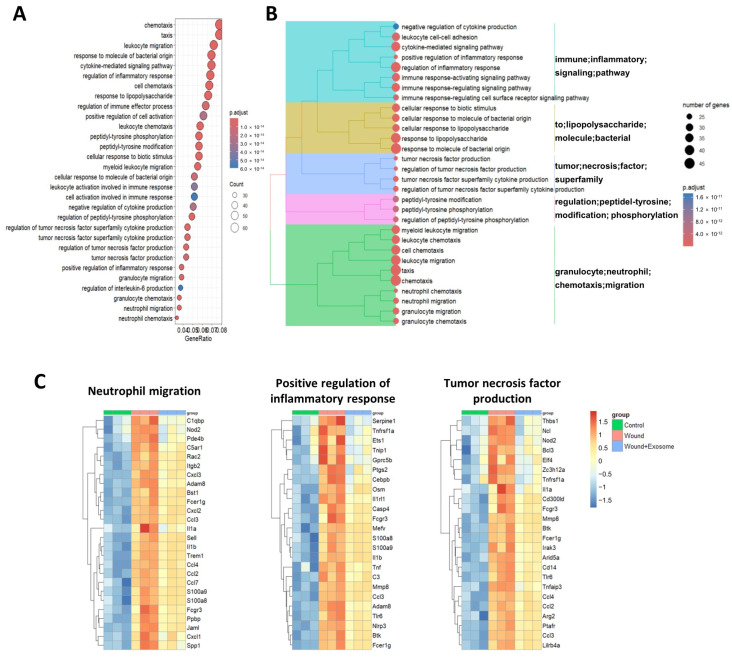
Profiles of the differentially expressed genes (DEGs) suppressed by the retinal organoid (RO)-derived exosome (Exo-RO) treatment in the corneal injury model. (**A**) List of the biological processes of the genes that were upregulated in the phosphate-buffered saline (PBS)-treated group compared with the controls, which were subsequently downregulated by the Exo-RO treatment. (**B**) Cluster analysis showing that these biological processes had distinct characteristics. (**C**) Representative processes that were significantly increased upon corneal injury, with subsequent downregulation after the Exo-RO instillation, including “neutrophil migration”, “positive regulation of inflammatory response”, and “tumor necrosis factor production”.

A subsequent GO enrichment analysis indicated that the 413 genes upregulated in the PBS-treated corneas and reversed by the Exo-RO treatment were predominantly enriched in various biological processes, such as chemotaxis, the cytokine-mediated signaling pathway, regulation of the inflammatory response, the response to lipopolysaccharides, and the positive regulation of cell activation (Figure 7A). Further categorization revealed distinct clusters among these terms (Figure 7B). The Kyoto Encyclopedia of Genes and Genomes (KEGG) pathway enrichment analysis showed that the 413 genes were enriched in pathways such as cytokine–cytokine receptor interactions, PI3K-Akt signaling, TNF signaling, MAPK signaling, and IL-17 signaling (Supplementary Figure S4). Figure 7C shows the altered gene expression of the mainly enriched GO terms and pathways (e.g., neutrophil migration, positive regulation of inflammatory response, and TNF production) between the experimental groups, indicating that the Exo-RO treatment suppressed inflammatory processes upon corneal injury.

**Figure 8:**
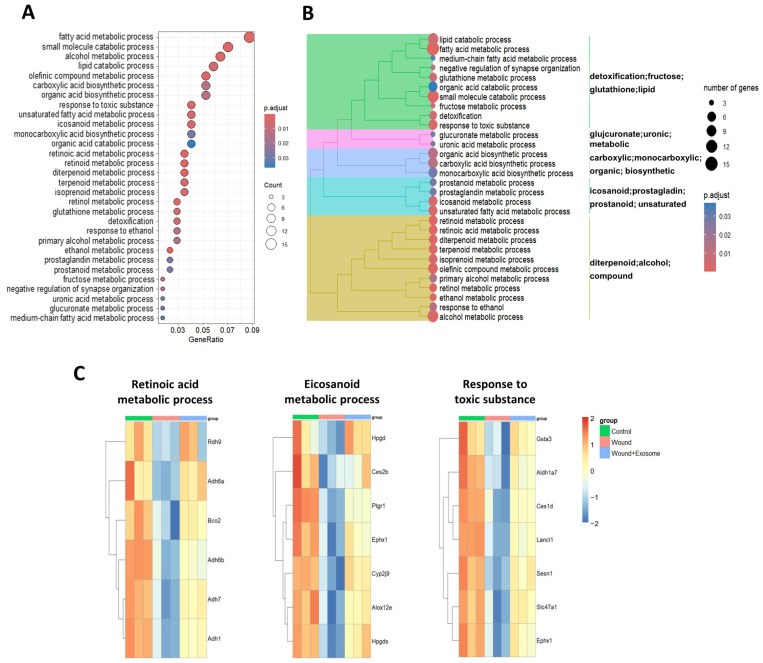
Profiles of the differentially expressed genes (DEGs) upregulated by treatment with the retinal organoid (RO)-derived exosomes (Exo-ROs) in the corneal injury model. (**A**,**B**) Gene ontology terms for the DEGs that were downregulated in the phosphate-buffered saline (PBS)-treated group compared with the controls, which were reversed with the Exo-RO treatment. (**C**) The representative biological processes of the DEGs activated by the Exo-RO treatment were “retinoic acid metabolic process”, “eicosanoid metabolic process”, and “response to toxic substance”.

In contrast, the GO enrichment analysis of the 204 genes which were downregulated in the PBS-treated group and subsequently restored by treatment with the Exo-ROs revealed their involvement in fatty acid metabolism, alcohol metabolism, olefinic compound metabolism, the response to toxic substance, eicosanoid metabolism, and the retinoid metabolic process (Figure 8A,B). The KEGG pathway enrichment analysis demonstrated that the 204 genes were enriched in pathways such as drug metabolism, glutathione, arachidonic acid, and retinol metabolism (Supplementary Figure S5). The gene expression patterns of the main enriched GO terms and pathways, including retinoic acid metabolism, eicosanoid metabolism, and the response to toxic substances, are presented in Figure 8C. Among the various eicosanoids, the genes related to the degradation of prostaglandins (PGs), as well as the production of 12- and 15-hydroxy/hydroperoxyeicosatetraenoic acids (HETEs) and epoxyeicosatrienoic acids (EETs), were significantly upregulated (Supplementary Figure S6), suggesting that the regulation of such substances resulted in enhanced corneal wound healing after the Exo-RO treatment.

## 3. Discussion

In this study, we successfully isolated exosomes from the culture medium of the hiPSC-derived ROs, as confirmed by TEM and Western blotting analyses. Using a mechanical corneal injury mouse model, we observed superior wound healing in the Exo-RO-treated eyes compared with the vehicle-treated eyes. Our analysis suggested that the treatment effect was due to the immunomodulatory and proliferative properties of the Exo-ROs. This study was the first to demonstrate that exosomes derived from hiPSC-ROs have therapeutic potential in corneal epithelial wound healing.

There are several advantages to using exosomes as therapeutic agents compared with the actual delivery of stem cells to an injured site. Exosomes do not create safety issues such as immunological rejection, malignant transformation, or abnormal cell proliferation; nevertheless, they provide the paracrine effects of stem cells for wound repair [14,15]. Due to their stable chemical properties, they can be safely stored for long periods and may exert long-lasting effects via their contents, including proteins, mRNAs, and miRNAs [9,16]. Finally, exosomes readily enter their target cells due to their small size, and they show short-term therapeutic effects at sites of injury.

The therapeutic effects of stem cell-derived exosomes on corneal epithelial wounds have been explored, mainly using MSC-derived exosomes. Samaeekia et al. [4] reported that MSC-derived exosomes were successfully internalized into wounded corneal epithelial cells, resulting in superior wound healing compared with a vehicle-treated cornea epithelium, both in vitro and in vivo. A recent study showed the therapeutic effects of exosomes derived from umbilical cord MSCs on corneal epithelial defects, demonstrating enhanced wound recovery through activation of the PI3K/Akt signaling pathway [17]. In the present study, we used exosomes derived from ROs after 50 to 60 days of differentiation as a possible therapeutic agent, considering that the retina and cornea both originate from ectoderm, and there is evidence of crosstalk (involving various transcription factors) between the neural retina and cornea development during early stages of eye development [18,19]. Moreover, we previously found that the miRNAs in Exo-ROs were mainly related to the MAPK and PI3K/AKT signaling pathways [20], which are expected to have beneficial roles in modulating inflammation, apoptosis, and cell proliferation/migration in the context of corneal injury [21,22].

Corneal epithelial cells are continuously regenerated from the limbus, where a rich population of limbal stem cells responds to heal corneal epithelial wounds [23]. When the corneal epithelium is damaged, the following three major repair mechanisms are involved in its regeneration: epithelial cell proliferation, cell migration from nearby basal epithelium, and cell differentiation into stratified layers [24]. Limbal stem cells, especially basal limbal epithelial stem cells, are reportedly responsible for centripetal migration along the basal membrane upon acute corneal injury [25]. In the present study, we observed that the Exo-RO treatment significantly enhanced epithelial cell proliferation throughout the injured cornea, from the limbus to the leading edge, and the greatest increase was evident in the limbus. Consistent with previous reports, the increase in proliferative activity was more intense in the basal epithelial layer, indicating that the enhanced wound healing achieved by the Exo-ROs was at least partially due to the increased proliferation and differentiation of basal limbal epithelial stem cells.

In addition to the increased proliferative activity, the Exo-ROs used in this study had immunomodulatory effects, as demonstrated by the qRT-PCR and exosomal miRNA sequencing, along with the total RNA sequencing of the treated corneal tissues. During epithelial regeneration, complex interactions among various cytokines, growth factors, chemokines, neuropeptides, and matrix metalloproteinases are responsible for wound healing [26,27]. Dysregulation of this process may hinder wound healing and lead to permanent corneal stromal scarring, and the suppression of pro-inflammatory factors can improve wound healing. In the present study, treatment with the Exo-ROs resulted in the suppression of well-known inflammatory cytokines and chemokines involved in wound healing, including TNF-α, CCL2, and CCL5. TNF-α can physically disrupt the corneal epithelial barrier under stress conditions [28,29] and delay corneal epithelial cell migration, leading to impaired wound healing [30]. CCL2 and CCL5 are monocyte chemoattractants that strongly induce monocyte recruitment and migration to injured or infected sites [31,32,33]. Overexpression of these chemokines upon acute injury may lead to an excessive inflammatory response, resulting in permanent corneal opacity. Suppression of these cytokines by the Exo-ROs may have accelerated the corneal epithelial wound healing with a relatively transparent underlying stroma, as was observed in the present study.

Our total RNA sequencing analysis revealed the upregulation of genes related to retinoic acid, fatty acid, and eicosanoid metabolic processes in the Exo-RO treated corneas. These processes are major regulators of tissue repair/regeneration and the inflammatory response. Retinoic acid, a small biologically active molecule derived from vitamin A (retinol), is essential for corneal repair and regeneration [34,35], whereas fatty acids and eicosanoids have distinct roles in corneal wound repair. Among the fatty acids, cyclooxygenase products such as PGs may exert harmful effects on epithelial healing, whereas lipoxygenase derivatives (e.g., 12-HETEs, 15-HETEs, and EETs) promote epithelial proliferation and repair [36]. Our analysis showed that the genes related to the enzymes that degrade PGs and produce 12-HETEs, 15-HETEs, and EETs were upregulated after the Exo-RO treatment, revealing that the regulation of fatty acid production may be another important mechanism underlying the enhanced corneal wound healing we observed after the Exo-RO treatment. The upregulated gene expression related to “response to toxic substance” and “organic acid biosynthetic process” may have further promoted the corneal wound healing. Additional studies are needed to elucidate the complex underlying mechanisms related to these processes in corneal epithelial wound repair and to investigate the feasibility of developing potential therapeutic targets based on the signaling pathways revealed in this study.

This study showed that treatment with Exo-ROs promoted corneal epithelial wound healing. Such therapeutic effects were achieved by increased epithelial cell proliferation and migration from the stem cells at the limbus, along with significantly suppressed inflammatory processes and the regulation of substances related to wound healing (e.g., retinoic acid and eicosanoids). These findings suggest that Exo-ROs can serve as a novel therapeutic agent for impaired corneal epithelial wound healing.

## 4. Materials and Methods

### 4.1. Animals

Eight-week-old male C57BL/6J mice (Orient Bio Inc., Seongnam, Republic of Korea) were used for the experiments. The animals were housed in a temperature- and humidity-controlled room with a 12 h light/12 h dark cycle, and they were provided food and water ad libitum. All animal experiments were conducted in accordance with the Guide for the Care and Use of Laboratory Animals and the Association for Research in Vision and Ophthalmology Statement for the Use of Animals in Ophthalmic and Vision Research, and all animal studies were approved by the Institutional Animal Care and Use Committee of Soonchunhyang University Hospital, Bucheon, Republic of Korea (approval number: SCHBCA 2023-05). The mice were sedated with an intraperitoneal injection of a mixture of 40 mg/kg of zolazepam/tiletamine (Zoletil^®^ 50; zolazepam/tiletamine 25 mg/25 mg/mL, Virbac, Carros, France) and 5 mg/kg of xylazine (Rompun; xylazine 100 mg/mL, Bayer Healthcare, Leverkusen, Germany) before any surgical procedures.

### 4.2. hiPSC Culture into Three-Dimensional ROs

RO differentiation was conducted using American Type Culture Collection (ATCC)-DYR0100 hiPSCs (ACS-1011; ATCC, Manassas, VA, USA) for 60 days, as previously described [37]. The details and the timelines of the RO differentiation are presented in the Supplementary Experimental Methods and in Supplementary Figure S1.

### 4.3. Exosome Isolation and NTA

For the exosome isolation, 1 mL of medium containing five ROs at days 50 to 60 was collected to isolate the exosomes secreted from the ROs. The collected medium was centrifuged at 3000× *g* for 10 min to remove cell debris and then frozen at −80 °C until further analysis. The exosomes were extracted from the cultured retina organoid medium using a miRCURY exosome isolation kit (Qiagen, Hilden, Germany), in accordance with the manufacturer’s instructions. Briefly, after the conditioned medium had been filtered with a 0.22-µm syringe filter, corresponding amounts of reagents were added in proportion to the medium volume. The mixtures were vortexed and incubated at 4 °C overnight and then centrifuged at 10,000× *g* for 30 min at 20 °C; next, the exosome pellet was resuspended in 100 μL of manufacturer-supplied suspension buffer for a 1 mL starting medium volume. All exosomes were stored at –80 °C immediately after isolation and maintained at that temperature until further use.

NTA was performed to determine the concentrations of Exo-ROs using a NanoSight NS300 instrument equipped with NTA 3.4 analytical software and a 488 nm laser (Malvern Panalytical, Malvern, UK). At least five 30 s videos were recorded for each sample in light scatter mode with camera levels of 11 to 13, and we used the same software settings for all measurements (screen gain 10 and detection threshold 7). Before the analysis, the exosome samples were diluted to an appropriate concentration in 0.22 µm filtered PBS.

### 4.4. Exosome Identification: TEM and Western Blotting

TEM images of the exosomes derived from the ROs were obtained. A 1:10 dilution of the exosome suspension in PBS was incubated on formvar/carbon-coated, charged nickel grids (200 mesh; Electron Microscopy Sciences, Hatfield, PA, USA) for 2 min. The grids were then fixed with 2.5% glutaraldehyde for 10 min, washed with 0.1 M PBS, and blotted. Next, the grids were placed on 8 µL of 2% uranyl acetate for 2 min. The grids were then examined and imaged using a JEM-1400 flash electron microscope equipped with an AMT XR401 sCMOS camera and AMT Capture Engine software (version 7.00; JEOL Ltd., Tokyo, Japan).

For the Western blotting, the exosome pellets were resuspended in lysis buffer containing 10 mM of HEPES (pH of 7.9), 0.1 mM of EDTA, 0.1 mM of EGTA, 5% glycerol, 1 mM of DTT, and 400 mM of NaCl; an RO whole-cell lysate was used as a control. Protein samples of the lysates (5 μg/well) were boiled in sodium dodecyl sulfate (SDS) loading dye for 5 min and separated by 9% SDS–polyacrylamide gel electrophoresis. The protein samples were then transferred to PVDF membranes. After the membranes had been blocked with 5% skim milk for 1 h and washed with 0.1% Tween-20 in Tris-buffered saline, they were incubated overnight with antibodies against CD9 (Cat#13174; Cell Signaling Technology, Danvers, MA, USA) and CD63 (Cat#sc-365604; Santa Cruz Biotechnology, Dallas, TX, USA) as positive markers for the exosomes [13] and β-actin (Cat#A1978; Sigma-Aldrich, St. Louis, MO, USA) and calnexin (Cat#2679; Cell Signaling Technology) as negative markers for the exosomes [13]. After the membranes had been washed with PBS containing Tween-20, they were incubated for 1 h with the appropriate peroxidase-conjugated anti-mouse or rabbit IgG (1:5000; Santa Cruz Biotechnology). Immunoreactivity was visualized using a chemiluminescent reagent (Atto, Tokyo, Japan) and an Azure imaging system (Azure Biosystems, Dublin, CA, USA). The intensities of the bands were quantified using ImageJ software (version 1.54g; National Institutes of Health, Bethesda, MD, USA).

### 4.5. Development of the Corneal Injury Model and Exosome Instillation

The mice were deeply sedated and administered a topical anesthesia using 0.5% proparacaine; then, an approximately 2 mm central epithelial defect was produced using a sterile disposable biopsy punch (Kai Medical, Tokyo, Japan). Immediately after the injury, the corneas were instilled with 5 μL of an exosome suspension containing 1.0 × 10^6^ exosomes/μL, or PBS as the vehicle control at 0, 10, and 20 min. Wound healing at 0, 12, 24, and 36 h was monitored by fluorescein staining, and the epithelial defect was photographed using a camera-equipped biomicroscope. Wound-healing ratios (%) were calculated by comparisons with the baseline epithelial defect sizes for each mouse using ImageJ software (version 1.54g) [38].

### 4.6. Exosome Uptake Detection

The exosome uptake by the corneal epithelium was detected by staining the exosomes with calcein (Calcein-AM; Thermo Fisher Scientific, Waltham, MA, USA), a green fluorescent dye. Briefly, the exosomes (100 μL) were incubated with 1 mM of calcein-AM for 30 min at 37 °C in the dark. This mixed suspension was resuspended in 1 mL of PBS, and the calcein-tagged exosomes were extracted using an exosome isolation kit (Qiagen), in accordance with the manufacturer’s instructions. To examine the exosome uptake by the corneal epithelium in vivo, the calcein-labeled Exo-ROs (5 μL) were instilled in injured and uninjured mouse corneas. Three hours after instillation, the corneal tissues were harvested to examine the uptake of the instilled Exo-ROs. A Z-stack of 25 μm corneal sections was photographed using a laser confocal microscope (Leica DMi8; Leica Microsystems, Wetzlar, Germany). Further experiments to observe the therapeutic effects of the Exo-ROs were conducted using the unlabeled exosomes.

### 4.7. 5-ethyl-2′-deoxyuridine Assay

To analyze the epithelial cell proliferation, the mice were intraperitoneally injected with EdU (100 mg/kg) immediately after the injury and treatment with either the Exo-ROs or the vehicle. At 24 h after injury and treatment, the mice were deeply anesthetized and intracardially perfused with 0.1 M of PBS containing 150 U/mL of heparin, followed by 4% paraformaldehyde (PFA) in 0.1 M of PBS. Next, the eyes were enucleated, and 360-degree sclerotomies were performed immediately behind the limbus to separate the lenses and posterior segments from the anterior segments. The tissues were fixed with 4% PFA, incubated in 30% sucrose in PBS overnight, and embedded in an optimal cutting temperature compound. Serial 10 mm sections were cut and mounted on adhesive microscope slides (HistoBond; Marienfeld-Superior, Lauda-Königshofen, Germany). The corneal sections were washed with 3% bovine serum albumin in PBS for 2 min and then with PBS for 2 min. The proliferation activity was assessed with an EdU assay using Click-iT EdU Imaging kits (Thermo Fisher Scientific, Waltham, MA, USA) [39]. The nuclei were visualized by Hoechst 33342 staining (1:2000) in PBS for 2 min at room temperature. The tissue sections were then washed, sealed with coverslips using mounting medium (Dako, Santa Clara, CA, USA), and imaged with a confocal microscope (Leica DMi8; Leica Microsystems, Wetzlar, Germany).

### 4.8. Quantitative Reverse Transcription-Polymerase Chain Reaction

The mouse corneas at 36 h after treatment were used for the isolation of the total RNA with TRIzol^®^ reagent (Thermo Fisher Scientific), and first-strand cDNA was synthesized using a SuperScript™ III First-Strand Synthesis System (Thermo Fisher Scientific) in accordance with the manufacturer’s protocol. The cDNA was amplified using a QuantiSpeed SYBR Hi-ROX Kit (PhileKorea, Seoul, Republic of Korea) on a StepOnePlus™ Real-Time PCR System (Applied Biosystems; Thermo Fisher Scientific). The thermocycling conditions were as follows: polymerase activation at 95 °C for 2 min, followed by 40 cycles of 95 °C for 5 s (denaturation) and 60 °C for 30 s (annealing/extension). A melt curve analysis was performed at 95 °C for 15 s, 60 °C for 1 min, and 95 °C for 15 s for each step. Table 1 lists the primers used. GAPDH was used as an internal control. The mRNA expression levels were quantified using the 2^−ΔΔCT^ method.

### 4.9. RNA Sequencing

The total and exosomal RNA were extracted using TRIzol reagent (Thermo Fisher Scientific) in accordance with the manufacturer’s instructions. The RNA quality was assessed with an Agilent 2100 bioanalyzer using an RNA 6000 Pico Chip (Agilent Technologies, Amstelveen, The Netherlands), and the RNA was quantified using a NanoDrop 2000 Spectrophotometer (Thermo Fisher Scientific).

An exosomal miRNA library was constructed using a NEBNext Multiplex Small RNA Library Prep kit (New England Biolabs, Ipswich, MA, USA) in accordance with the manufacturer’s instructions, which are described in detail in the Supplementary Experimental Methods. The miRNA target genes were predicted using miRTarBase v8.0 and TarBase v8.0 in miRNet (https://www.mirnet.ca (accessed on 29 July 2024)). For the GSEA to predict the functions of the exosomal miRNAs related to inflammation [40], we transferred the gene sets containing the terms “inflammatory”, “cell adhesion”, or “epithelial cell” in the biological processes of the GO database (http://geneontology.org/ (accessed on 29 July 2024)) to a gmt file containing the miRNA sets that were predicted to be the target genes in each GO (Supplementary Table S4). The gmt file was used for the preranked GSEA test for the exosomal miRNAs and sorted in descending order of the normalized cpm values.

A total RNA sequencing library was prepared using a NEBNext Ultra II Directional RNA-Seq Kit (New England Biolabs). The mRNAs were isolated using a Poly(A) RNA Selection Kit (LEXOGEN, Inc., Vienna, Austria). The isolated mRNAs were used for the cDNA synthesis and shearing, in accordance with the manufacturer’s instructions (details in Supplementary Experimental Methods). The processed high-throughput sequencing reads were mapped to the reference genome using HISAT2 (http://daehwankimlab.github.io/hisat2/main/ (accessed on 29 July 2024)), and the read count was extracted using HTseq [41]. The data were processed based on the FPKM+Geometric normalization method, a differentially expressed gene (DEG) analysis, and a generalized linear model quasi-likelihood F-test using EdgeR within R (R development Core Team; The R Foundation for Statistical Computing, Vienna, Austria) [42]. The EnhancedVolcano (Bioconductor, Seattle, WA, USA; https://bioconductor.org/packages/EnhancedVolcano (accessed on 29 July 2024)) and pheatmap in R (The R Foundation for Statistical Computing; https://CRAN.R-project.org/package=pheatmap (accessed on 29 July 2024)) packages were used to visualize the results of the DEG analysis. The target genes of the up- or down-regulated miRNAs were obtained using the multiMiR package [43]. GO and KEGG pathway enrichment analyses were performed using the clusterProfiler package [44] with the default settings. The similarities of the obtained GO or KEGG pathways were calculated and visualized using the enrichplot package (Bioconductor, Seattle, WA, USA; https://bioconductor.org/packages/EnhancedVolcano (accessed on 29 July 2024)).

### 4.10. Statistical Analysis

All data are shown as means ± standard errors, and the Student *t*-test was used to identify the significant differences among the groups. All experiments were independently repeated at least three times. Statistical analyses were conducted using SPSS for Windows (version 20.0; SPSS Inc., Chicago, IL, USA). Differences with *p*-values of < 0.05 were considered statistically significant.

## Figures and Tables

**Table 1:** Primers used in this study.

Gene	Forward	Reverse	Transcription ID
TNF-α	TCAAACCCTGGTATGAGCCC	ACCCATTCCCTTCACAGAGC	ENSMUST00000025263.15
CCL2	AGGTCCCTGTCATGCTTCTG	TCTGGACCCATTCCTTCTTG	ENSMUST00000000193.6
CCL5	ACTCCCTGCTGCTTTGCCTAC	GAGGTTCCTTCGAGTGACA	ENSMUST00000035938.3

## Data Availability

Data will be made available on request.

## Supplementary Materials

The following supporting information can be downloaded at: https://www.mdpi.com/article/10.3390/ijms25168925/s1.
