# Supplementary material for: Exosomes from Human iPSC-Derived Retinal Organoids Enhance Corneal Epithelial Wound Healing
Source: Int J Mol Sci. 2024 Aug 16;25(16):8925. doi: 10.3390/ijms25168925 (PMC11354741; doi:10.3390/ijms25168925)
Supplement: Supplementary file 1 [file ijms-25-08925-s001.zip › Supplementary materials_final_revised (1).pdf]

Supplemenatary materials

Exosomes from Human iPSC-derived Retinal Organoid Facilitates Corneal Epithelial Wound Healing

Si Hyung Lee, Jung Woo Han, Jin Young Yang, Jungmook Lyu, Hyo Song Park, Byung Soo Kim, Ji Hong Bang, Yeji Kim, Hun Soo Chang, and Tae Kwann Park

Supplementary experimental procedures

hiPSC Culture into three-dimensional ROs

The hiPSCs were maintained on vitronectin coated culture dish with E8 media and dissocaited by treatment with ReLeSR (STEMCELL Technologies, Vancouver, Canada). Dissociated cells were plated on a low attachment 6-well plate containg E8 medium with 3uM ROCK inhibitor Y27632 (Tocris Biosciences, Abingdon, UK) and 3uM Blebbistain (Tocris Biosciences, Abingdon, UK) at day 0 to induce Embryoid bodies (EB) formation. EB were gradually replaced by neural induction medium (NIM) containing DMEM/F12 (1:1, Gibco, Grand Island, NY), 1% N2 supplement (Gibco, Grand Island, NY), non-essential amino acids, and 2 µg/ml heparin (STEMCELL Technologies, Vancouver, Canada) from E8 medium without Y27632 and Blebbistain. Day of detachment was annotated as day 0, with the medium being changed on day 1 (25% NIM), day 2 (50% NIM) and day 3 (100% NIM). On day 7, EB were plated on 35mm matrigel-coated dish (Corning, Tewksburty, MA) containing NIM at a density of 150 EB per dish. On day 15, the medium was switching from NIM to retinal differentiation medium (RDM), consist of DMEM/F12 (3:1), 2% B-27 supplement without vitamin A (Gibco, Grand Island, NY), NEAAs, and antibiotic-antimycotic, and were change every other day. On day 25-28, the loosely adherent central portions of the neural clusters were lifted using a P1000 pipettor under Evos XL cell iamging microscope (Invitrogen, Waltham, MA). The picked optic vesicle-like aggregates were futher cultured to form three-dimensional retinal organoids with RDM, was supplemented with 10% exosome-depleted FBS (Gibco, Cat. No. A2720801), 100 mM Taurine (Sigma Aldrich, St. Louis, MO), and 2 mM GlutaMAX (Thermo Fisher Scientific). The medium was changed every 3 days until the 60 days of RO differentiation (Supplementary Figure S1).

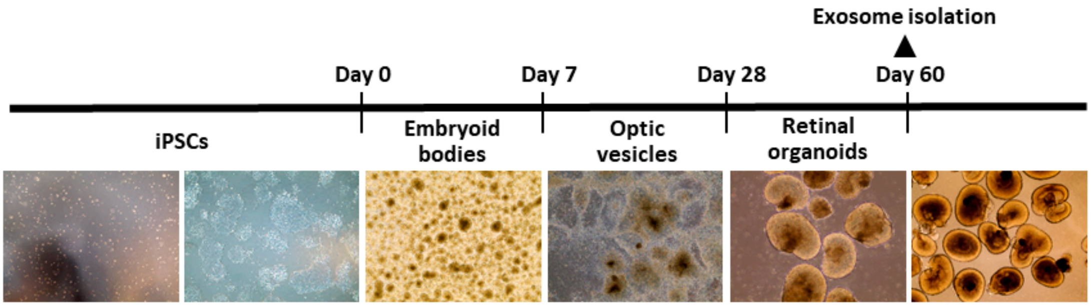

Supplementary Figure S1. hiPSC-derived Retinal Organoids (ROs).

Timeline of RO differentiation protocol showing main steps during RO differentiation. Exosomes were isolated on day 60 of differentiation from RO conditioned media.

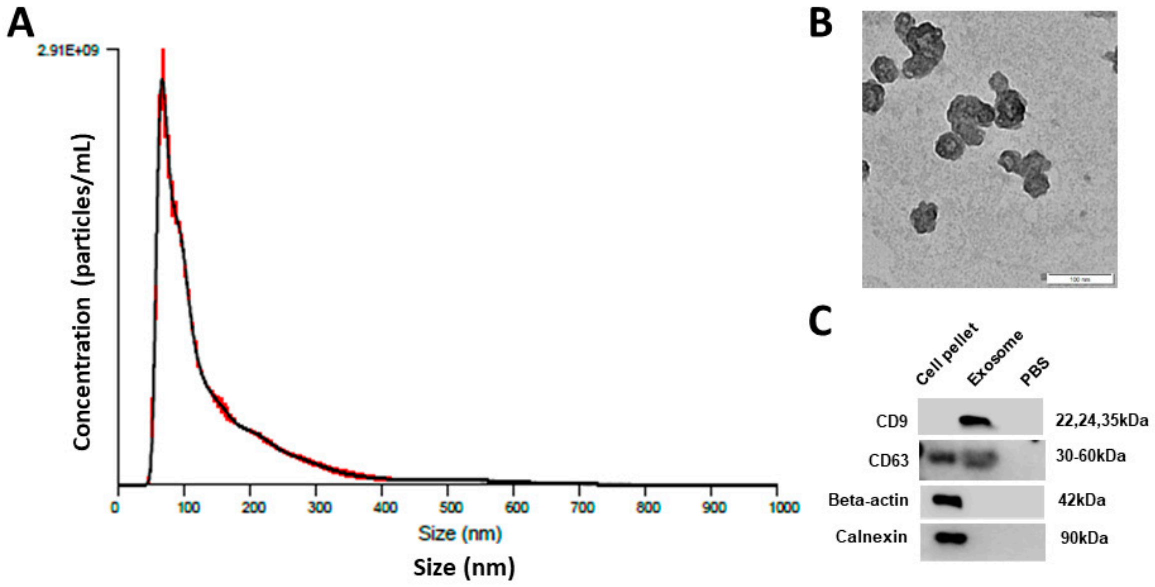

Supplementary Figure S2. Characteristics of exosomes derived from ROs.

(A) Distribution of size and concentration and (B) transmission electron microscopy images of exosomes isolated from hiPSC-derived ROs. (C) Western blot analysis on the expression of the exosomal markers. Scale bar : 100nm.

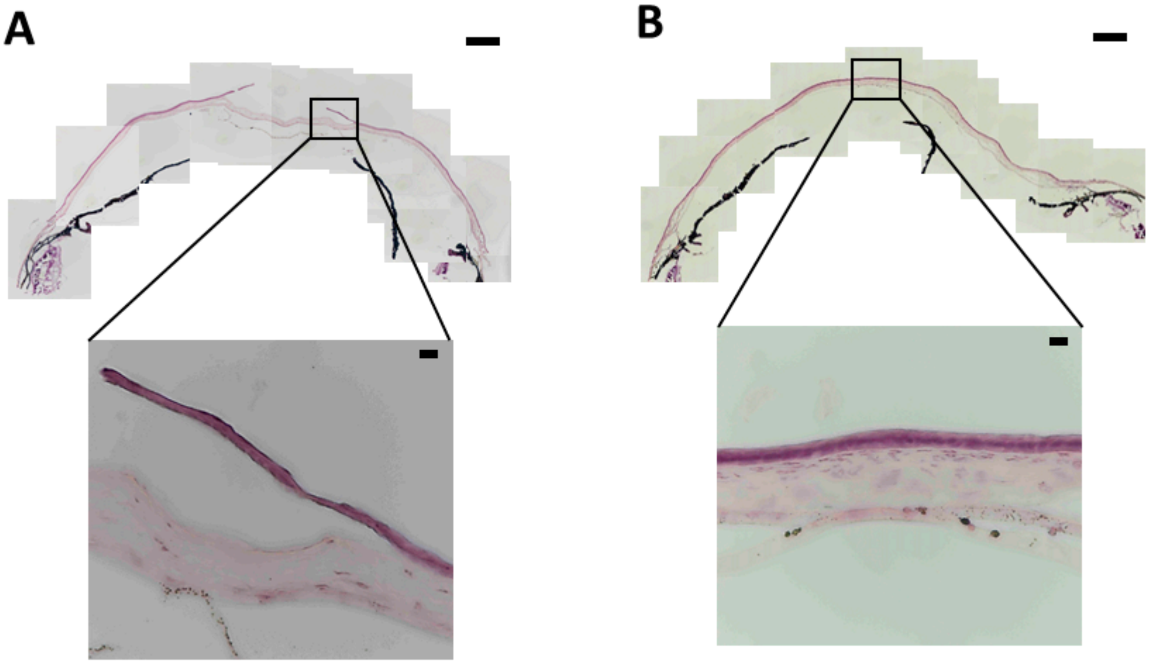

Supplementary Figure S3. Hematoxylin-eosin (H&E) stained images of corneas treated with PBS and Exo-ROs.

(A) Images of H&E staining of PBS-treated injured cornea and (B) Exo-ROs treated cornea after 36 hrs of treatment. Below images are magnified images from center of corneas. Scale bar : 250μm.

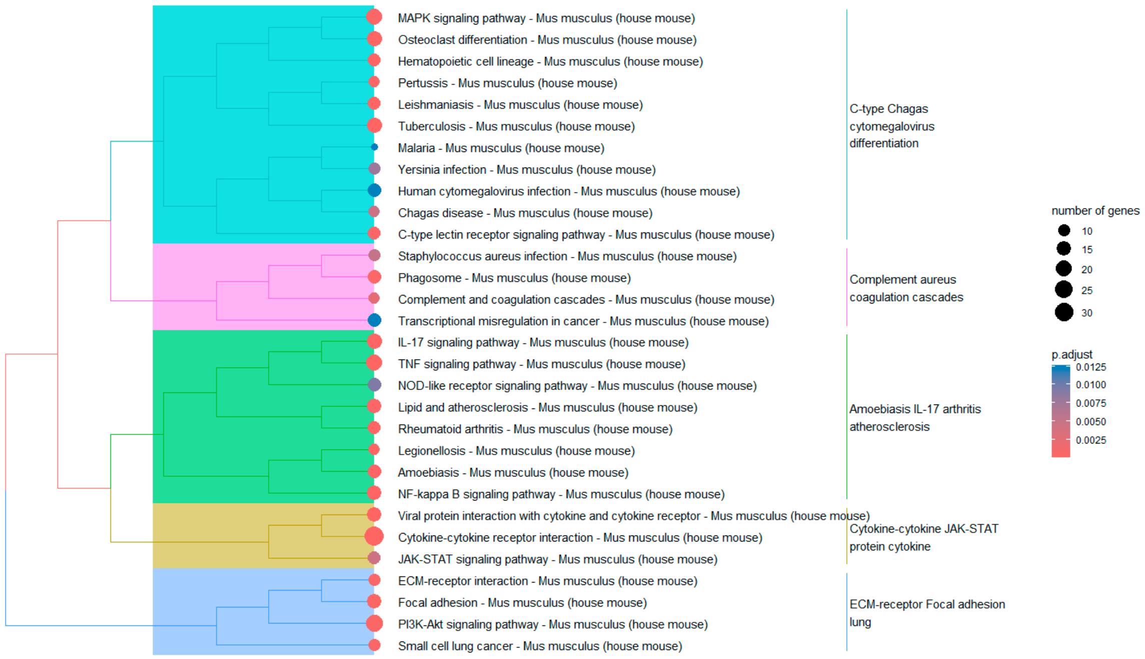

Supplementary Figure S4. KEGG pathway enrichment analysis was conducted on 413 genes that were upregulated in the corneal wound and subsequently reversed by RO-Exo treatment.

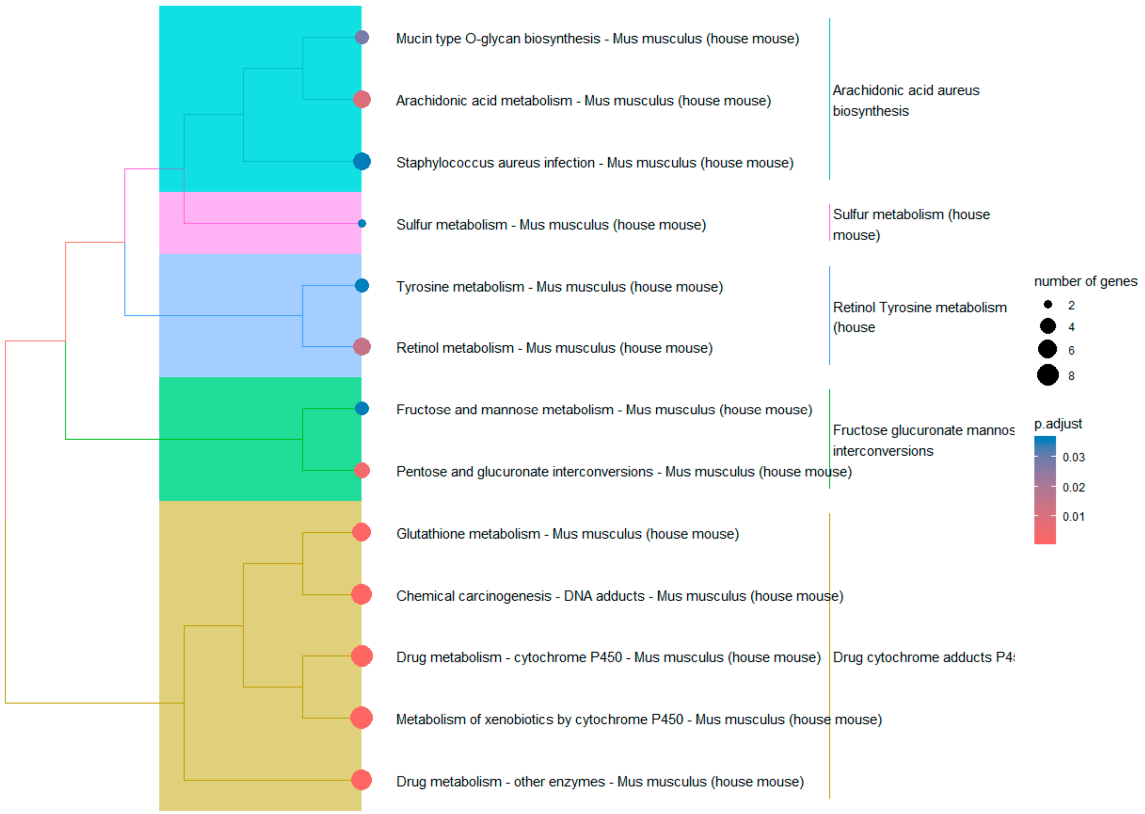

Supplementary Figure S5. KEGG pathway enrichment analysis was conducted on 204 genes that were downregulated in the corneal wound and subsequently restored by RO-Exo treatment.

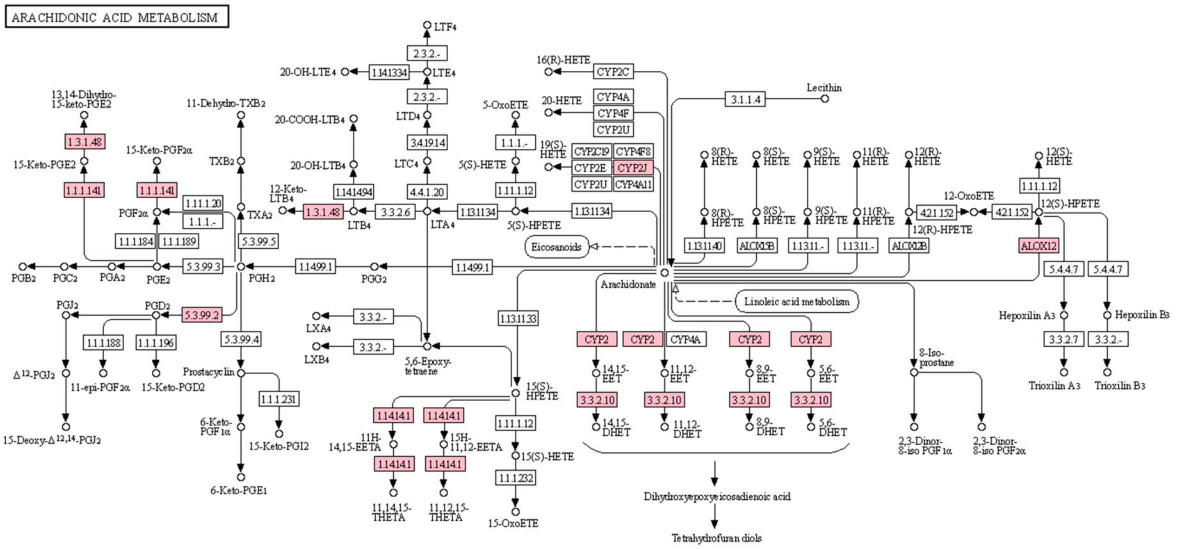

Supplementary Figure S6. The Kyoto Encyclopedia of Genes and Genomes pathway for arachidonic acid metabolism. Genes with significantly different expression after Exo-RO treatment are highlighted with red.
